# Differential expression analysis with global network adjustment

**DOI:** 10.1186/1471-2105-14-258

**Published:** 2013-08-23

**Authors:** Jonathan A Gelfond, Joseph G Ibrahim, Mayetri Gupta, Ming-Hui Chen, Jannine D Cody

**Affiliations:** 1 Department of Epidemiology and Biostatistics, University of Texas Health Science Center San Antonio, San Antonio, Texas, USA; 2 Department of Biostatistics, University of North Carolina School of Public Health, Chapel Hill, North Carolina, USA; 3 School of Mathematics and Statistics, University of Glasgow, Glasgow, Scotland, UK; 4 Department of Statistics, University of Connecticut, Storrs, Connecticut, USA; 5 Department of Pediatrics, University of Texas Health Science Center San Antonio, San Antonio, Texas, USA

## Abstract

**Background:**

Large-scale chromosomal deletions or other non-specific perturbations of the transcriptome can alter the expression of hundreds or thousands of genes, and it is of biological interest to understand which genes are most profoundly affected. We present a method for predicting a gene’s expression as a function of other genes thereby accounting for the effect of transcriptional regulation that confounds the identification of genes differentially expressed relative to a regulatory network. The challenge in constructing such models is that the number of possible regulator transcripts within a global network is on the order of thousands, and the number of biological samples is typically on the order of 10. Nevertheless, there are large gene expression databases that can be used to construct networks that could be helpful in modeling transcriptional regulation in smaller experiments.

**Results:**

We demonstrate a type of penalized regression model that can be estimated from large gene expression databases, and then applied to smaller experiments. The ridge parameter is selected by minimizing the cross-validation error of the predictions in the independent out-sample. This tends to increase the model stability and leads to a much greater degree of parameter shrinkage, but the resulting biased estimation is mitigated by a second round of regression. Nevertheless, the proposed computationally efficient “over-shrinkage” method outperforms previously used LASSO-based techniques. In two independent datasets, we find that the median proportion of explained variability in expression is approximately 25%, and this results in a substantial increase in the signal-to-noise ratio allowing more powerful inferences on differential gene expression leading to biologically intuitive findings. We also show that a large proportion of gene dependencies are conditional on the biological state, which would be impossible with standard differential expression methods.

**Conclusions:**

By adjusting for the effects of the global network on individual genes, both the sensitivity and reliability of differential expression measures are greatly improved.

## Background

A goal of systems biology is to understand how a perturbation affects a network of interrelationships between genes. There are many models for gene networks, but few have shown accurate predictions across many datasets [1]. There is a growing collection of gene perturbation experiments in which a subset of transcipts’ expression have been modulated, either through carcinogenesis, chemical treatments, or through siRNA [2]. These experiments comprise an information-rich dataset that allows us to construct global network models and test their predictive accuracy on the level of the full transcriptome.

Network models can be helpful for distinguishing between all differentially expressed genes and the genes that are immediately affected by a perturbation such as a gene deletion. There are clinically relevant reasons for this line of investigation. For instance, chromosomal abnormalities are the most common cause of mental retardation in the US, and the deletion of a chromosomal segment is a common subtype [3]. The deleted segments contain many genes, so that this is a type of sporadic, multiple gene knockout. The deletions are often *hemizygous*; that is, only one of the two (maternal or paternal) homologous chromosomes with the segment remains intact. Hence, the genes within the deleted segments are present with half of the normal copy number. Most genes exhibit some form of dosage-sensitive decrease in expression (e.g., DNA deletion tends to decrease mRNA expression), but there is a possibility that the gene’s regulatory network can compensate for the loss by selectively increasing the expression of the intact copy. The ability or inability of the regulatory networks to compensate with the intact copies on the homologous chromosome could explain which genes are responsible for common abnormal phenotypes. In this paper, we examined subjects with nearly identical somatic deleted segments of the q-arm of chromosome 18, and we performed comparative gene expression microarrays of these subjects with a parental control sample. Our goal is to: 1) identify the global gene expression differences, 2) to identify which genes are the most affected by the global expression differences relative to their regulatory networks, and 3) to identify genes that have different regulatory networks in case and control groups.

Various methods have been developed both for predicting gene expression and adjusting for the correlations between genes in differential expression analyses. Dahl et al [4] improved the detection of differential expression by grouping genes into co-expression clusters with a Dirichlet process mixture model. Leek at al [5] proposed a general framework for significance testing when a large-scale number of components have mutual dependency. This method may be applied to the differential expression problem and allows independent testing of hypotheses by conditioning on an orthogonal dependence kernel. These methods have shortcomings because they do not explicitly utilize the vast amount of data from prior experiments.

Some network methods do use extensive prior data such as Ruan et al [6] who used the cluster average of a set of co-expressed genes as a prediction for another gene within the cluster. This relatively simple method had similar or superior accuracy to models that used substantial auxiliary data including regulatory pathways and DNA-binding patterns of transcription factors such as the approach taken by Gustafsson et al [7] that used an “elastic-net" penalty [8] and information from multiple genomic modalities (microarray, ChIP-seq, etc.). Along the lines of Ruan et al [6], we present a simple and computationally efficient method for predicting gene expression. Our method extends the work of Congrove et al [9] who modeled a gene’s expression as linearly dependent on other genes, but we have added features that improve computational efficiency and robustness to variations in transcriptional networks.

## Methods

Suppose we have a series of microarrays represented by a matrix *Y* that is *G* × *N* where *G* is the number of the genes on the *N* arrays, and the expression measurements for each gene *g* will be the row vector *y*_*g*_, *g* ∈ [1 … *G*]. We define the perturbation vector *X* as an *N* × 1 vector with elements *x*_*i*_ = *I*(*i*^*th*^array is perturbed) where *I*(·) is the indicator function. Our goal is to estimate the effect of the perturbation conditional on the gene expression network *P*(*y*_*g*_|*X*,*Y*_−*g*_) where $Y_{-g}=\{y_{g^{\prime }}:g^{\prime }\neq g,g^{\prime }\in \;[1\ldots G]\}$. That is, we want to predict the effects of the perturbation on a gene given that it is within a regulatory network consisting of all other genes. Typically, the sample size (*N*) of the experimental data *Y* is small ≤10, but we assume that a database of experiments *Y*_*D*_ (*G* × *N*_*D*_) is also available with a substantially larger sample size *N*_*D*_ ≥ 100. Our method will use this database *Y*_*D*_ as an independent training set to estimate the regulatory network within *Y*.

We make the simplifying assumption that the log-transformed gene expression *y*_*g*_ follows an approximately Gaussian distribution with mean *β*_*g*_*Y*. A predictive model for each gene within *Y* and *Y*_*D*_ can be constructed by using Sparse Simultaneous Equations Model (SSEM) [9,10]. Under this model, the gene expression of a transcript *y*_*g*_ can be estimated by a weighted sum of the remaining transcripts. The key parameters are within *β*_*g*_ (1 × *G*) where the $\beta_{gg^{\prime }}$ elements are the influence that gene *g*^′^ has on gene *g* and represent the overall transcription network with *β*_*gg*_ = 0 so that genes do not influence themselves. These *β*_*g*_ parameters can be estimated from a database of related array experiments. Thus, we have the model

(1) $$y_{g}=\beta_{g}Y+\varepsilon_{g}$$

where *ε*_*g*_ is an 1 × *N* vector of Gaussian errors. This may be expressed in matrix form as

(2) Y=BY+E

where *B* is *G* × *G* and composed of the $\beta_{gg^{\prime }}$ parameters such that *B* has diagonal elements consisting of zeros, and *E* is the *G* × *N* matrix of Gaussian errors with rows *ε*_*g*_. The intrinsic gene network can be described by *B*. Cosgrove et al [9] model the effect of the perturbation of this network by introducing *ϕ*_*g*_

(3) $$y_{g}=\beta_{g}Y+\phi_{g}+\varepsilon_{g},$$

and in matrix form

(4) Y=BY+Φ+E.

The Φ parameter is the *G* × *N* matrix composed of *ϕ*_*g*_ and is the *direct* effect of the perturbation *X* on gene expression that is not accounted for by the gene network. Their goal is to estimate these direct actions Φ upon gene expression to elucidate, for example, the direct drug targets. They estimated the *B* matrix by using *compendia* or a large (*N* > 100) database of experiments *Y*_*D*_, and estimate Φ using the residuals

(5) $$r=y^{\text{pert}}-By^{\text{pert}}=\phi^{\text{pert}}+\varepsilon^{\text{pert}}.$$

Cosgrove et al [9] used *ϕ*^*pert*^ as the estimator of the direct action of the perturbation. Further, they did not take into account that the network *B* may not be the same in different cell types or biological conditions, which we consider in our approach below.

Although it was originally applied to large genomic databases, this model can be extended to apply to situations where the current experiment has insufficient data in order to accurately estimate the *B* parameter. Furthermore, estimating *B* with a large database *Y*_*D*_ and applying it to a new dataset will avoid the problem of using the data twice. For instance, if we have a large database of expression measures *Y*_*D*_, we may estimate *B*_*D*_ such that *Y*_*D*_ = *B*_*D*_*Y*_*D*_ + *E*, under the assumption that *B*_*D*_ ≈ *B*. Given our estimate of *B*_*D*_ with rows *β*_*Dg*_, we may then construct a linear predictor of each gene *g* in the new dataset *Y* by *Y*_N*g*_ = *β*_*Dg*_*Y*.

With a covariate matrix *X* that applies to the individual samples, we propose the model

(6) $$y_{g}=\beta_{\text{Dg}}Y\nu_{g}+X^{\prime }\delta_{g}+\varepsilon =Y_{Ng}\nu_{g}+X^{\prime }\delta_{g}+\varepsilon .$$

This equation differs from Equation 3 as *β*_*Dg*_*Yν*_*g*_ = *Y*_N*g*_*ν*_*g*_ is substituted for *β*_*g*_*Y* and *X*^′^*δ*_*g*_ is substituted for *ϕ*_*g*_. The additional *ν*_*g*_ scalar parameter represents a very important increase in model flexibility compared to Cosgrove et al [9] where *ν*_*g*_ is constrained to be 1.0. In the new approach, *ν*_*g*_ is estimated for each gene so that if the historical network is inconsistent with the current network and is not a good predictor for gene expression in the current experiment, then its effect on inference approaches zero as *ν*_*g*_ does. The effect of the biological condition on gene *g* is described by the scalar *δ*_*g*_, and this is distinct from the usual formulation of differential expression that tests the effect of *X* (*N* × 1) alone on *y*_*g*_[11,12]. The *δ*_*g*_ parameter corresponds to an element of the Φ parameter, but unlike *ϕ*_*g*_ in Equation 3, *δ*_*g*_ is *jointly estimated* with *ν*_*g*_ and undergoes formal statistical testing in our approach rather than acting merely as a basis for ranking candidates. Hence, the hypothesis *H*_0_ : *δ*_*g*_ = 0 is the test of differential expression, conditional on the value of the historical gene network. The p-value can be derived through standard linear model theory, and the status of differential expression can be determined by setting a cutoff for p-values or false-discovery rates. The use of the database *Y*_*D*_ to construct *Y*_N*g*_ (1 × *N*) is key because it allows the network effect within *Y* to be estimated with only one additional unknown parameter *ν*_*g*_, which is essential for datasets with relatively few biological replicates. It is noteworthy that the differential expression hypothesis using Equation 6 is an increase (or decrease) in a gene’s expression conditional on the gene-network, which is a somewhat different hypothesis than standard differential expression. Additionally, we further extend the previous model of [9] by considering interactions between the network prediction and the covariate matrix by defining the 1 × *N* element-wise multiplicative interaction term (*Y*_N*g*_ × *X*) within

(7) $$y_{g}=Y_{Ng}\nu_{g}+X^{\prime }\delta_{g}+(Y_{Ng}\times X)\gamma_{g}+\varepsilon .$$

The tests of the scalar *γ*_*g*_ = 0 correspond to the test of disruption of the gene network associated with the covariate *X*. By modeling the disruption of the network, we may test biologically interesting hypotheses and further improve the accuracy of predicting gene expression.

Even with large gene expression databases, regularization is required for accurate parameter estimation due to the condition *N* ≪ *G* because of the large number of parameters in the model for each gene. The prior model [9] used the Lasso [13] that has an *L*_1_ penalty and produces a parsimonious model with many regression coefficients close to 0. We observed that the Lasso produced unstable regression coefficients with split sample training and validation sets, but prediction accuracy was nevertheless maintained. In a parsimonious model, some transcripts from a set of correlated transcript predictors would have non-zero coefficients whereas the other transcripts in the set would have coefficients close to zero. In this manner, the predictive weight would be unevenly shared by the correlated transcripts. However, Ruan et al [6] obtained good success in predicting gene expression by using a k-nearest-neighbor (KNN) method. The KNN method used the average of co-expressed genes as a predictor, which gives equal predictive weight to a set of correlated transcripts. We suggest a balance between unequal, *parsimonious* (Lasso) and equal, *robust* (KNN) weight among correlated transcript predictors. One such compromise is the elastic net that combines penalties on the *L*_1_ and *L*_2_ norms. The elastic net, however, carries a substantial burden in computational cost and risk of overfitting by optimizing over two parameters.

We offer a more efficient compromise and call our proposed approach Over-Shrinkage Ridge Regression (OSRR). The OSRR model is fit using standard ridge regression (RR) and ordinary least squares (OLS) implementations. The gene expression values from the database *Y*_*D*_ and the new data of interest *Y* are all centered at 0, which is numerically convenient. The gene expression variances are not scaled to all have equal variance because the scale of variability is biologically relevant. We are applying the network estimates from one array platform to another, which are on the log-scale, and we assume that fold-changes (i.e., a doubling of gene-expression) will be meaningful across platforms. After centering, the first step of the model fit is the estimation *β*_*Dg*_ with penalty *λ* based upon the database *Y*_*D*_. In this approach, the regularization penalty takes the same form as standard ridge regression, and the first objective function to be minimized for each gene is

(8) $$∥y_{\text{Dg}}-\beta_{\text{Dg}}Y_{D}{∥}^{2}+\lambda ∥\beta_{\text{Dg}}{∥}^{2}$$

where ∥·∥^2^ is the Euclidean norm. The *β*_*Dg*_ are the ridge regression coefficients with *y*_*Dg*_ as the outcome and $Y_{D,-g}^{\prime }$ (*Y*_*D*,−*g*_ = *Y*_*D*_without row *g*) as the covariate matrix. The identifiability of *β*_*Dg*_ when *λ* > 0 follows from the theory of ridge regression [14]. As *λ* → *∞*, the regression parameter estimates *β*_*Dg*_ will tend towards 0 as the penalty becomes the dominant term. Hence, the larger the *λ*, the more the *β*_*Dg*_*Y*_*D*_ is biased towards the null compared to the observed database values *y*_*Dg*_. However, we introduce a second step and add another parameter *ν*_*g*_ to counteract the bias towards the null in predicting the data from a small experiment *Y* with rows *y*_*g*_. The second step is the estimation of *ν*_*g*_ conditional on the *β*_*Dg*_ that minimizes ∥*y*_*g*_ − *β*_*Dg*_*Yν*_*g*_ ∥^2^ using ordinary least squares. If we let *Y*_*Ng*_ = *β*_*Dg*_*Y*, then the estimation of *ν*_*g*_ can be seen as the univariate OLS coefficient of *Y* regressed onto *Y*_*Ng*_. That is, the second objective function operates on the new data *Y*, and the estimate of row *y*_*g*_ is *β*_*Dg*_*Yν*_*g*_ where *ν*_*g*_ minimizes

(9) $$∥y_{g}-Y_{\text{Ng}}\nu_{g}{∥}^{2}$$

using ordinary least squares. The introduction of *ν*_*g*_ within a second stage of the regression has two effects. First, $\beta_{g}^{\text{OSRR}}=\beta_{\text{Dg}}\nu_{g}$ is no longer biased towards the null as *λ* → *∞*. The larger *λ* might make *β*_*Dg*_*Y* smaller than *y*_*g*_ by an order of magnitude, which is the motivation for the term “Over-Shrinkage". Second, larger values of *λ* will act to equalize the elements of *β*_*g*_ corresponding to correlated transcripts. This equalization property produces a similar effect as KNN’s equal weighting, but OSRR still allows data-driven deviation from equality and allows genes that are not the nearest neighbors to influence prediction. Hence, we call this a global network adjustment. An important difference between OSRR and approaches such as ridge regression, LASSO, and the elastic net is that these methods penalize the magnitude of the parameter *β* norms with some function *P*(*β*) whereas the OSRR approach considers a link function between the linear predictor *Xβ* and the outcome *Y*. The link function *g*(*Xβ*) is the second regression so that the model becomes *Y* = *g*(*Xβ*), which may serve to minimize the effect of poor prediction.

The robustness of the OSRR method to variable gene regulatory networks and applicability to smaller sample sizes are its attractive features. If the gene expression network estimate *B*_*D*_ from *Y*_*D*_ results in a prediction *Y*_*Ng*_ that is uncorrelated with the smaller independent dataset *Y*, then the regression parameter *ν*_*g*_ converges to 0 under standard linear model theory. This property is quite distinct from the use of the LASSO model by [9] that does not consider larger training sets for estimating *B* or variable gene-gene dependencies due to the perturbation or biological state. The selection of *λ* is based upon the maximum correlation *across all genes* between the database prediction *β*_*Dg*_*Y* and the current experiment *Y*. Overfitting may result because the OSRR is applied to the same data it is tuned with (i.e., *λ* is selected based upon *Y*). OSRR largely avoids overfitting and gains simplicity by using a single tuning parameter *λ* for all transcripts. The method was implemented in the R statistical software [15], and we fitted the model in equation 6 with the lme4 R package [16]. A random intercept was used to account for the correlation within case-control pairs. The programs, example simulated data, and a demonstration of the penalty selection are available for download from the supplemental website as Additional file 1.

## Simulation studies

**Figure 1:**
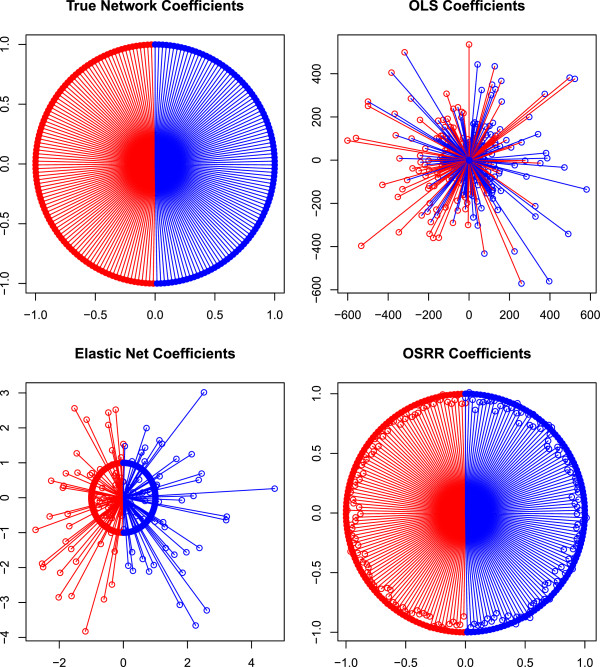
**Network depiction of regression estimation.** The regression coefficients are shown as rays from the origin. The absolute value of the coefficient is the distance from the origin and the sign is indicated by blue (+) or red (-). There are 200 rays indicating 200 estimated coefficients, and the true values of the coefficients (+1,-1) appear as rings. The Ordinary Least Squares (OLS) estimates are highly variable because of the small sample size and the correlation between the positive and negative predictor groups. The elastic net has less variable estimates than OLS, but the OSRR coefficients demonstrate the the strong smoothing property of that equalizes the influence of predictors.

We performed a simulation study to illustrate the properties of OSRR in the context of correlated covariates with similar effects on a continuous outcome. Here, we consider a single outcome *y* (*n* × 1) with two independent sets of correlated predictors *X*_1_ and *X*_2_ both with dimension (*N* × *G*). The *N* = 201 rows of *X*_1_ and *X*_2_ are drawn from a compound-symmetric, multivariate Gaussian distribution with dimension *G* = 100, mean 0 and variance 1.0 with correlation *ρ* = 0.9. Both *X*_1_ and *X*_2_ represent two correlated sets of genes that have an influence on the expression of the gene *y*. Within these sets, the influence of the genes is the same, which might reflect the activity of two pathways that are either associated with an increase or an inhibition of the expression of *y*. That is, *y* = *X*_1_*B*_1_ + *X*_2_*B*_2_ + *ε* where $B_{1}={\left[\;\beta_{1}\ldots \beta_{1}\right]}^{\prime }$ and $B_{2}={\left[\beta_{2}\ldots \beta_{2}\right]}^{\prime }$ are *G* × 1 with identical elements *β*_1_ = 1.0 and *β*_2_ = −1.0, respectively. The errors *ε* have a Gaussian distribution with variance 1.0. The data were fit using 3 models: ordinary least squares (OLS), Elastic Net, and the OSRR with *λ* = 10,000. The elastic net penalty was selected using K = 5 fold cross-validation. The results are shown in Figure 1, which is a network representation of the coefficient estimates. All models perform similarly in terms of prediction. The estimates of *B*_1_ and *B*_2_ are highly variable for OLS. The elastic net predicts as well as other models, but the estimated coefficients are highly variable with some coefficients clustered around 0. To offset the Lasso parameter estimates close to 0, the remaining coefficients are estimated to be larger in magnitude than the true values. The OSRR exhibits a strong smoothing property that equalizes the influence that each of the correlated predictors has, whereas the elastic net and the Lasso tend to reduce the number of influential predictors. This is an advantage in that it provides robustness, but when there are sparse predictors or there is sufficient sample size to estimate the coefficients more precisely, then we anticipate that the OLS, elastic net or Lasso are advantageous.

**Figure 2:**
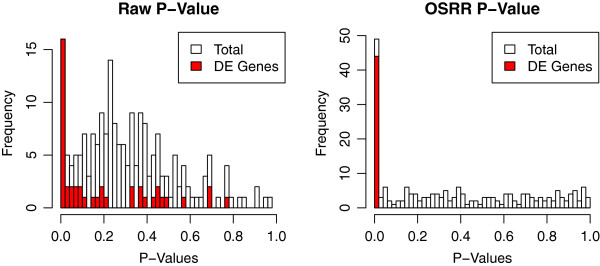
**Proof-of-concept simulated differential expression experiment.** Histograms of p-values are shown for both differentially expressed genes (DE) and not DE genes that follow the null hypothesis. The raw (naive) model has p-values for not DE genes that do not have the expected uniform distribution, though the p-values corresponding to the differentially expressed (DE) genes tend to be low. The OSRR model applied to the same dataset more clearly separates the DE from the not DE genes, and the distribution of the not DE genes is more uniform.

As a proof of principle, we performed another simulation study to demonstrate the advantage of the OSRR methodology applied to differential expression compared to a naive approach that does not account for prior knowledge about correlations between transcripts. We simulated a database *Y*_*D*_ with *G* = 200 and *N* = 100. The transcripts were given a block diagonal correlation structure with 2 blocks of size 100 transcripts each with compound symmetry having variance 1 and correlation *ρ* = 0.8. Although this is a relatively simple multivariate Gaussian model compared to actual gene networks, it still represents a large dependency between genes. The OSRR model was fitted as described to estimate *B*_*D*_ with *λ* = 1000 selected out of the set {10^2^,10^3^,10^4^,10^5^,10^6^} based upon the out-sample prediction from training and validation sets. We then simulated a smaller (*N* = 20) dataset *Y* with the same correlation structure. Ten of the arrays were under the control condition (*x*_*i*_ = 0), and 10 were under a treatment condition (*x*_*i*_ = 1). Differential expression was simulated by randomly selecting genes with probability 0.2 and then adding or subtracting *δ*_*g*_ = 1.0 to those genes. We compared the performance of the model in equation 6 to a model without correction for the network relationships *Y*_N*g*_. This naive model reduces to the t-test. The results are shown in Figure 2. The histograms for p-values corresponding to the null hypothesis (*δ*_*g*_ = 0) are given for both models, and the OSRR model clearly has smaller p-values for the differentially expressed (DE) genes compared to the naive model. Also, the naive model p-values do not have a uniform distribution for the not differentially expressed genes as the OSRR does. The lack of uniform distribution of the null p-values is due to the dependence structure and could inflate the Type I error. This is consistent with higher sensitivity and specificity of OSRR relative to naive methods that do not utilize prior information on gene networks.

## Empiricial studies

### Chromosomal deletion study

In our chromosome deletion study, we investigated the effects of 18q chromosomal deletions (deleted segments were the 60,021,550−76,117,153 bps of chromosome 18) on cell lines derived from blood samples called lymphoblastoid cell lines (LCLs). There were 10 case-control (c-c) pairs with the case subject having a deletion on chromosome 18 (18q-), and the control was a normal genotype, same-sex parent giving a total of *N* = 20 = 2 × 10 subjects. We assayed each c-c pair with 4 Agilent 44K expression arrays. We used a dye-swap design such that for 2 arrays the case was labeled with Cy3 (control labeled with Cy5) and 2 with case labeled with Cy5 (control labeled with Cy3). Each case had associated clinical phenotypes including height, weight, age, and various laboratory measures of growth hormone responsiveness. The log-expression data for a given subject and gene are denoted as *y*_*grs*_ where the indices *g*, *r*, and *s* represent gene, replicate, and spot respectively. The Cy3 and Cy5 channels were treated separately, and we did not use the log-ratio. For each subject, we averaged the replicates and the spots corresponding to the same gene yielding the *N* × 1 vector *y*_*g*_. We did not model the dye factor because it was balanced and not predictive. There is a corresponding *N* × 1 covariate matrix *X* where *x*_*i*_ = *I*(*i*^*th*^subject is a case). We also examined the p-values of the *δ*_*g*_ parameter from the model compared to a standard analysis with the LIMMA software [12].

For *Y*_*D*_, we used a much larger dataset from [17] who studied similar LCL cells that were treated with a variety of chemotherapies. There were a total of 374 Affymetrix chip assays in this dataset. We included all genes that were within the deleted region, but otherwise restricted our analysis to genes that had gene symbols in common to both the Affymetrix and the Agilent platforms and were expressed in the Affymetrix data. This leaves a total of 5,035 genes to estimate *B*_*D*_. The expression values of the database genes were centered, but not scaled. A key issue is how to select the penalty parameter *λ*, and the selection process can be extremely computationally intensive as [9] used 200 CPU days for their cross-validation Lasso approach. To greatly simplify the selection of the penalty and minimize the tuning and hypothesis testing on the same dataset, we chose one penalty parameter for all genes and took a split sample approach so that the database was divided into two sets of 187 arrays. Further, we took into account three aspects of goodness-of-fit in assessing cross-validation. First, we considered prediction among the split samples and second, we considered the stability of the regression parameters *B*_*D*_ between the split samples. Third, we examined the prediction of *B*_*D*_ with respect to our independent experiment. The computation time for selecting *λ* took approximately 151,445 CPU seconds (8GB RAM, 2 Ghz) or about a 100 fold decrease in computing time relative to the Lasso approach for a similar sized dataset and computing environment [9]. The Based upon these three criteria, we selected *λ* = 10^4^ out of the set {10^2^,10^3^,10^4^,10^5^,10^6^}, and we applied the corresponding ${\hat{B}}_{D}$ to construct predictors $Y_{Ng}={\hat{\beta }}_{\text{Dg}}Y$ for *y*_*g*_ from our expression experiment. Lastly, to further assess the robustness and Type I error, we performed a permutation analysis comparing OSRR and a conventional method. We permuted the case-control labels 100 times, and compared the histograms of the p-values from both methods.

### Results

**Figure 3:**
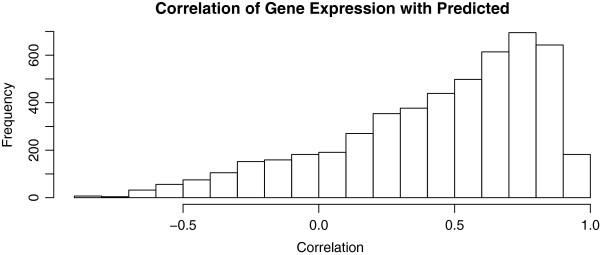
**Histogram of correlations of the predictor** ***Y***_**N*g***_** based upon regulatory network from database*Y***_***D***_** with gene expression in an independent experiment** ***Y*.** The positivity of the center of the distribution implies a dependency of gene expression that is consistent with the OSRR model prediction.

We observed that the network predictions *Y*_N*g*_ based upon ${\hat{B}}_{D}$ estimated from the database *Y*_*D*_ were strongly correlated with new data *y*_*g*_. In Figure 3, the median correlation of *Y*_N*g*_ with *y*_*g*_ is 0.54 so that $25\%\approx \sqrt{0.54}$ of the variance of transcription regulation is explained. Note that the correlation of the observed expression with the network predictor is expected to be > 0, and the mean correlation is significantly greater than 0 (*p* < 0.0001). The degree of prediction is remarkable because Cosgrove et al [9] originally studied bacteria, whereas these data indicate that more complex human transcriptional patterns even on different microarray platforms are largely predictable. Nevertheless, for some genes, there is a negative correlation consistent with random noise. This underscores the importance of the estimation of *ν*_*g*_ in equation 6. That is, if the network model does not fit the smaller dataset, then *ν*_*g*_ is estimated to be close to 0 so that its effects are mitigated.

**Figure 4:**
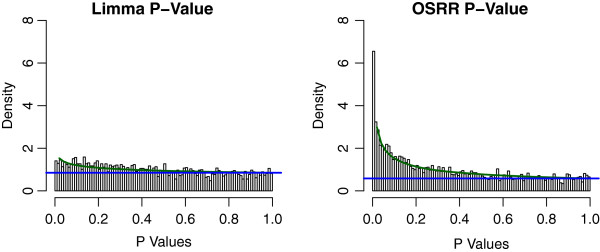
**Histogram of p-values generated by conventional LIMMA and OSRR methods applied to the chromosomal deletion study.** Note that LIMMA produces p-values that differ only slightly from a uniform distribution, but the OSRR method gives more significant p-values. The beta-uniform mixture model is shown in green and the uniform component is blue. The ratio of blue/green is the empirical Bayes probability of the false positive rate at a given p-value. This ratio approaches 0.5 for conventional analysis and it approaches 0.25 for the OSRR method.

Figure 4, shows the distribution of p-values for differential expression of transcripts under both the standard LIMMA analysis and OSRR models. Under the null hypothesis p-values are uniformly distributed between 0 and 1. The p-values of the LIMMA model do not appear to be for from uniformly distributed, but the OSRR model not only has more significant (*p* < 0.05) values, it also has a clear “spike" of small p-values towards 0. It is possible to model these types of distributions with a beta and uniform mixture (BUM) as suggested by Pounds et al [18] where the beta component models the spike of p-values that corresponds to differentially expressed transcripts and the uniform component corresponds to the transcripts that follow the null hypotheses. The BUM fit is shown in Figure 4, and the ratio of the the uniform (blue) component over the mixture density (green) is the empirical Bayes probability estimate of the false positive rate. The comparison of numbers of genes that at given p-values cutoffs is shown in Table 1. Clearly, the OSRR model identifies more than double (851 vs. 337) the number of significant transcripts, and those transcripts selected by the LIMMA approach are also selected by OSRR at a rate of 95% (319/337). The correlation between the − log(p-values) from LIMMA and OSRR is 0.88, which indicates fairly good agreement in ranking the genes by probability of differential expression. The mean value for the false discovery rate (FDR) of the 21 genes in the deleted region in the chromosome according to LIMMA was 0.51 compared to the mean FDR of the q-value adjusted OSRR p-values of 0.17, indicating that the OSRR model is more likely to identify the deleted genes as differentially expressed.

**Table 1:** Number of significant transcripts in standard LIMMA and OSRR models at different p-value cutoffs

**Up/Down**	**P-Value**	**LIMMA**	**OSRR**	**Both**
Down	0.0500	94	209	92
	0.0100	25	79	25
	0.0010	9	19	9
	0.0001	5	8	5
Up	0.0500	241	642	227
	0.0100	46	251	42
	0.0010	6	52	5
	0.0001	2	15	2

Up/Down corresponds to higher/lower expression in the cases relative to controls.

Not only was the OSRR method shown to identify twice the number of differentially expressed genes, but the log fold-change estimates are more stable, and therefore less likely to be false positives based upon the split-sample analysis. We randomly split the data set 20 times into two sub-datasets of 5 families each, and compared the results of OSRR and the t-test from the two sub-datasets of the partition in estimating the log-fold change between cases and controls. Note that LIMMA is equivalent to the t-test in estimating log-fold change. Table 2 lists the correlations between the in-sample and out-sample comparisons of the differential expression estimates of the conventional model $\delta_{g}^{\text{Std}}$ and the OSRR model *δ*_*g*_. The OSRR estimate was much more reproducible than the conventional estimate in independent samples with correlations of 0.321 (OSRR) vs. 0.201 (Standard) (95% CI for difference [0.054,0.183], *p* < 0.001). Furthermore, the out-sample results clearly show that OSRR is more consistent with both OSRR and conventional estimates in independent split datasets.

**Table 2:** Reliability assessed by a repeated split-sample analysis

**In/Out Sample**	**Type**	**Mean correlation**	**Std Dev**
In-Sample	*δ*_*g*_ vs $\delta_{g}^{\text{Std}}$	0.917	0.034
Out-Sample	$\delta_{g}^{\text{Std}}$ vs $\delta_{g}^{\text{Std}}$	0.203	0.115
Out-Sample	*δ*_*g*_ vs *δ*_*g*_	0.321	0.073
Out-Sample	*δ*_*g*_ vs $\delta_{g}^{\text{Std}}$	0.313	0.089

The parameters *δ*_*g*_ and $\delta_{g}^{\text{Std}}$ denote the differential expression estimates for OSRR and standard methods.

We compared the performance of the elastic net predictions with OSRR using a subset of 500 genes because of the computational time of the elastic net in this context. We selected a gene-specific penalty as did Cosgrove et al [9] who used the Lasso. We compared the average correlation of the predictions with the expression of the 500 genes in our smaller dataset, and we found that the mean correlation was 0.01 95% CI [−0.01,0.03] (paired t-test: *p* = 0.43) higher for the OSRR model. This is consistent with OSRR allowing equal prediction at a fraction of the computational cost of the elastic net.

**Figure 5:**
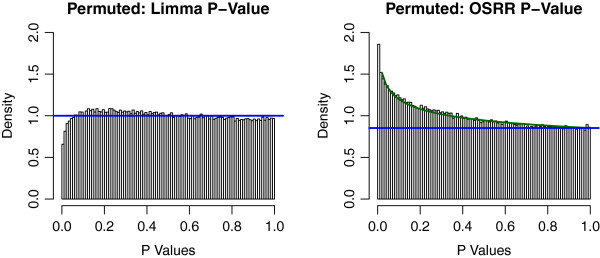
**Histogram of the p-values of the from the permuted chromosome deletion study.** The conventional analysis method has a slightly conservative, non-uniform null distribution. Whereas, the OSRR method has slightly anti-conservative, non-uniform null distribution.

The permutation study results are shown in Figure 5. The resulting estimates of the null distributions are different for the conventional and the OSRR model. The LIMMA model has a conservative Type I error rates indicated by nonuniform p-value distributions with a dip near 0, and the OSRR model has a slightly inflated Type I error with more smaller p-values than uniform. The OSRR model p-values are quite different from the non-permuted data in Figure 4. In contrast, the conventional analysis null distribution is more similar to the non-permuted distribution of p-values, which suggests a low signal-to-noise ratio. This is consistent with the OSRR model having a reasonably controlled Type I error rate, but much greater power to detect differences between the cases and controls.

We also applied the interaction model in Equation 7. A substantial proportion (10%, 484/5035) of genes had interactions (*H*_0_: *γ*_*g*_ = 0 with *p* < 0.01) between the dependence on the gene network and case-control status. This implies that the regulatory relationships are different for particular genes between cases and controls. The gene with the most significant interaction effect was the GARS gene, which has been linked to severe neurological conditions [19]. Interestingly, 93% of the 100 most significant interactions were negative ( ${\hat{\gamma }}_{g}<0$), which implies that the expression predictions are substantially less positively correlated with the observed expression in the abnormal cases than controls. See Additional file 2. This is consistent with overall disruption of the regulatory networks in the cases. Such observations would be impossible using standard differential expression models that only examine mean differences.

For the purpose of comparison, we applied the method of Cosgrove et al [9] to this dataset without the use of the larger training dataset using the elastic net estimators. Their method was not intended to be applied to datasets of this size (2 conditions (case/control) and 10 paired samples), but we wanted to investigate the necessity of using the database in a real example. Despite 5-fold cross-validation for the selection of the tuning parameters, there was overfitting of the gene network so that the estimates *BY* from the fit had a median correlation with *Y* of > 0.95 and the analysis of the residuals suggested in Equation 5 yielded p-values that did not differ from a uniform distribution (Data not shown). No differentially expressed gene could be identified due to the confounding of the gene expression network *BY* and the case-control status. However, we emphasize that their method was not intended for small datasets.

### Circadian rhythm study

**Figure 6:**
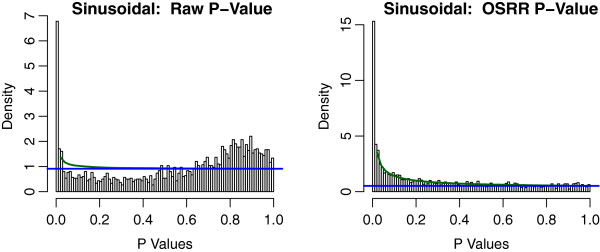
**Histogram of p-values generated by conventional** ***raw* and OSRR method of the circadian rhythm study.** The beta-uniform mixture model is shown in green and the uniform component is blue. The OSRR model identifies approximately double the number of significant genes.

**Figure 7:**
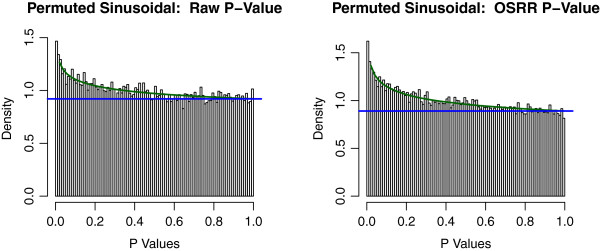
**Histogram of the p-values of the from the permuted circadian rhythm study.** Both the conventional analysis and the OSRR method have slightly non-uniform null distributions.

As a proof of principle, we considered another dataset to see if the OSRR model had similar advantages in a mouse model. This dataset [20] consisted of a time-series of brain tissue collected in mice with two different genotypes (wild type and a mutation of the circadian rhythm “clock" gene). The brain tissue samples were measured on Affymetrix tissue arrays in duplicate every two hours for 44 and 24 hours for a total of 24 and 14 arrays, respectively, for wild type and mutant mice. We estimated the *B* matrix using a database *Y*_*D*_ from an experiment with the same Affymetrix platform with 122 samples of mixed tissue types [21]. We assessed the 3,000 genes with the highest variance in the circadian dataset. We fit a sinusiodal model with and without network adjustment term *Y*_N*g*_*ν*_*g*_ to identify genes associated with the circadian rhythm

(10) $$y_{g}=Y_{Ng}\nu_{g}+a_{g}\;\cos\;(T+b_{g})+d+\varepsilon$$

where *T* (1 × *N*) is the time covariate in radians, *d* is an intercept, and the time dependencies are tested with scalars *a*_*g*_ and *b*_*g*_ (*H*_0_ : *a*_*g*_ = *b*_*g*_ = 0) using a log-likelihood test assuming that *ε* is Gaussian noise. We did not use the information about the genotype of the mice within the models. Rather, we tested whether or not the OSRR model could account for the biological variation due to different genetic backgrounds and still identify time dependent genes. The results are shown in Figures 6 and 7. Figure 6 shows the unpermuted data analysis in both models with the OSRR model having a higher proportion of significant genes. Figure 7 shows the null distributions under permutations, and the null distributions of the two models are very similar. This indicates that the OSRR model has more power to detect time-dependent genes with a similar Type I error rate as the conventional model. We also compared the performance of the elastic net and the OSRR gene expression predictions by the average correlations of the network predictors *Y*_N*g*_ with the outsample gene expression *y*_*g*_ for a random subset of 200 genes. We found that the OSRR method had a 0.1 higher average correlation (95% CI [0.05,0.15] with a paired t-test: *p* = 0.0003). That is, the OSRR had better prediction that the elastic net model for less computational costs; however, this advantage in prediction may be due to the smaller sample size of the training set (*N* = 122) relative to the chromosomal deletion training set (*N* = 374) for which the prediction of the two methods was approximately equivalent.

## Discussion

We have utilized the estimates of gene-networks from large databases in the analysis of small, independent datasets assayed on a different microarray type to successfully predict about 25% of the variation in transcript expression. The prediction is shown to markedly increase the sensitivity and the reliability of detecting differentially expressed transcripts in two different datasets. These inferences are different from the standard differential expression analyses because they reflect an adjustment based upon a regulatory model for each gene, and by testing an interaction term, one may make a statistical assessment of whether the regulatory network has changed between biological states. This method is approximately 100 times computationally faster than the previously reported method [9]. The majority of the computational time is spent fitting predictive models for each gene independently, and this time is multiplied by the density of the grid of the tuning parameter(s). OSRR has the advantages that the tuning parameter grid can be sparse and that the algorithms for fitting linear models are relatively well-optimized. The computation can be accelerated by parallelizing the fit, but parallel analyses are nontrivial because of large memory requirements of the operations (i.e., solving linear equations with 5,000 variables). Attention should also be paid to improving the computational efficiency of more complex models such as the elastic net.

Unlike the previous model, the use of the network predictor as a covariate in the differential expression regression model also provides robustness against poor prediction of particular transcripts. Given OSRR’s ease of implementation and its robustness, there is a broad set of potential applications to small sample size expression experiments that leverage the growing large-scale gene expression databases such as the Gene Expression Omnibus (GEO) [22]. We recommend that researchers select relevant datasets to construct network models by considering the species, type of tissue or cells, the microarray platform, and other sources of variation. We have seen that if the database *Y*_*D*_ used to estimate *B*_*D*_ has a different tissue type than the independent dataset *Y*, then the method tends to be less effective in accounting for variation.

## Conclusion

We have developed a novel adaptation of ridge-regression called OSRR that robustly estimates models of transcriptional co-expression networks based upon large microarray experiments. There are many possibilities for future research. Because of the robustness across microarray platforms, the OSRR approach can applied to RNA sequencing (RNA-Seq) data as well. In preliminary studies, we have found that the predictions of OSRR derived from microarray data perform similarly when applied to RNA-seq data. As we suggested with Equation 7, we can consider tissue specific modulations of regulatory networks as an extension of the OSRR model. Different tissue types or experimental conditions may induce different correlations between genes, and this fact is utilized in the COXEN model in [23] for disease classification purposes. These authors found that genes have different correlations in different cell types, and that genes that have shared correlation structures between two subtypes can predict how those subtypes will respond to chemotherapy. Also, the steps in the two-step fit are suggestive of levels within a hierarchical model. We can use this framework to extend the prediction model by including extra terms. For example, some genes may be better predicted using another network model with prediction *Y*_N2 *g*_. To account for this, we propose

(11) $$y_{g}=Y_{Ng}\nu_{Ng}+Y_{\text{N2}g}\nu_{\text{N2}g}+X\delta_{g}+\varepsilon .$$

If the other network model is a superior predictor, then this will be reflected by the *ν*_N2 *g*_ parameter. However, the more terms within the model, the more biological replicates are required for model stability. We may include information a known network of specific genes in a similar manner. We could further address another aspect of the SSEM problem. That is, the structure among the rows of *B* was not modeled. For example, if a gene had a relatively large influence on many other genes, then one could use this pattern for better estimation of *B*. This may potentially be achieved using a similar approach of Friedman et al [24] in the construction of the graphical Lasso.

## Competing interests

All of the authors deny competing interests, financial or otherwise, related to this research.

## Authors’ contributions

JAG helped conceive the methodology and performed the data analysis and simulation studies. JGI, MG, and MHC helped conceive and refine the methodology. JDC conceived and conducted the chromosomal deletion experiment and assisted in the interpretation of the experiment. All authors read and approved the final manuscript.

## Supplementary Material

Additional file 1 File with link to R implementation programs. Click here for file

Additional file 2 File with estimation results from chromosome 18 study for alterations in gene networks in cases relative to controls. Click here for file
